# γ-Aminobutyric Acid Suppresses Iron Transportation from Roots to Shoots in Rice Seedlings by Inducing Aerenchyma Formation

**DOI:** 10.3390/ijms22010220

**Published:** 2020-12-28

**Authors:** Changhua Zhu, Qi Qi, Huijiao Niu, Jiaqi Wu, Na Yang, Lijun Gan

**Affiliations:** College of Life Sciences, Nanjing Agricultural University, Nanjing 210095, China; zch@njau.edu.cn (C.Z.); 2018816118@njau.edu.cn (Q.Q.); 13135403358@126.com (H.N.); 23217212@njau.edu.cn (J.W.); yangna@njau.edu.cn (N.Y.)

**Keywords:** γ-aminobutyric acid, iron translocation, aerenchyma formation, iron-deficiency-related genes

## Abstract

γ-Aminobutyric acid (GABA) is a widely distributed non-protein amino acid mediated the regulation of nitrate uptake and Al^3+^ tolerance in plants. However, there are few reports about the involvement of GABA in the regulation of iron (Fe) acquisition and translocation. Here, we show that GABA regulates Fe homeostasis in rice seedlings. Exogenous GABA decreased the chlorophyll concentration in leaves, with or without Fe supply. Over-expression of glutamate decarboxylase (*GAD*) gene, coding a crucial enzyme of GABA production, elevated endogenous GABA content and caused more leaf chlorosis than wild type (Nipponbare). GABA inhibited Fe transportation from roots to shoots and GABA application elevated the expression levels of Fe deficiency (FD)-related genes under conditions of Fe-sufficiency (FS), suggesting that GABA is a regulator of Fe translocation. Using Perls’ blue staining, we found that more ferric iron (Fe^3+^) was deposited in the epidermal cells of roots treated with GABA compared with control roots. Anatomic section analysis showed that GABA treatment induced more aerenchyma formation compared with the control. Aerenchyma facilitated the oxidization of soluble ferrous iron (Fe^2+^) into insoluble Fe^3+^, resulted in Fe precipitation in the epidermis, and inhibited the transportation of Fe from roots to shoots.

## 1. Introduction

Iron (Fe) is an important micro-element for growth and development in plants, as it is required in many processes, including photosynthesis and respiration [1,2]. Although the earth is rich in Fe, it is scarcely available to plants, especially in alkaline soils, due to the formation of insoluble ferric iron (Fe^3+^) precipitates, which results in Fe deficiency (FD) [1,2]. FD is one of the largest problems in crop cultivation, as it limits plant productivity. On the other hand, higher concentrations of ferrous iron (Fe^2+^) present a risk, especially when plants are cultivated in acidic conditions [3,4]. Thus, an understanding of the mechanisms of Fe acquisition and translocation in plants is critical for the breeding of crops with better growth under different Fe conditions.

Plants have two ways for Fe uptake, referred to as strategies Ⅰ and Ⅱ. Non-graminaceous monocots and dicots use strategy Ⅰ, and graminaceous monocots use strategy Ⅱ, to deal with FD in soil [1,2]. Ferric reductase, iron-regulated transporters (IRTs), and acidification of the rhizosphere are involved in root Fe uptake employing strategy Ⅰ. In contrast, graminaceous plants produce the natural Fe^3+^ chelators mugineic acid (MAs) family phytosiderophores to solubilize Fe^3+^ [1,2]. In rice, MAs are secreted from roots to the rhizosphere via an exporter TOM1 [5], and the Fe^3+^–MA complex is then absorbed into the roots by yellow stripe/yellow stripe 1-like (YSL) transporters, such as OsYSL15 [6,7]. Research has shown that FD induces MAs accumulation, and that nicotianamine synthase (NAS), nicotianamine aminotransferase (NAAT), and deoxymugineic acid synthase (DMAS) are responsible for MAs biosynthesis from S-adenosylmethionine (SAM) [1]. All MAs share this synthetic pathway to deoxymugineic acid (DMA), which is then used to produce other MAs [8].

Previous studies have shown that citrate, nicotianamine (NA), and DMA are vital regulators in Fe transport through xylem [9]. In addition, YSL can also regulate the Fe transportation in the plant body, for example, OsYSL2 is a transporter of Fe(II)-NA and is involved in transportation of Fe in phloem [10]. Rice plants in submerged paddy fields also possess an Fe^2+^-transporter system, and several genes have been identified associated with Fe^2+^ transportation, such as *OsIRT1*, *OsIRT2*, and *OsNRAMP1* [11,12,13].

γ-Aminobutyric acid (GABA) is a widely distributed non-protein amino acid found in nature [14]. GABA concentrations have been found to increase as part of plant responses to adverse abiotic stresses, such as drought, anoxia, heat stress, salt, and low temperature [15,16]. Glutamate decarboxylase (GAD) is responsible for the conversion of glutamate to GABA [17]. GABA shunt involving the metabolism of GABA to succinic semialdehyde and then succinate and fed into the tricarboxylic acid cycle also affect GABA level in plant [18]. The half-life of D_6_-GABA was reported to be 1.3 h and it can be quickly converted to succinate [19].

GABA is also responsible for regulating the responses of plants to toxicity of metal elements, such as the possible regulation of arsenite, cadmium and aluminum tolerance [20,21,22]. Treatment with GABA abolished aluminum tolerance in wheat roots by inhibiting malate efflux [20]. The report of a GABA-mediated aluminum-activated malate transporter channel in plants provides further proof that GABA is a signaling molecule [16,23]. We recently reported that exogenous GABA treatment alleviated ammonium toxicity in rice seedlings under excessive NH_4_^+^ conditions by limiting NH_4_^+^ accumulation and assimilation [24]. However, few investigations on the function of GABA in Fe homeostasis in rice.

In this study, we investigated the effect of GABA on iron homeostasis in rice seedlings. The results will enhance our understanding of the function of GABA in plants and provide useful information on the mediation of Fe homeostasis in rice.

## 2. Results

### 2.1. GABA Participates in the Regulation of Iron Homeostasis

**Figure 1:**
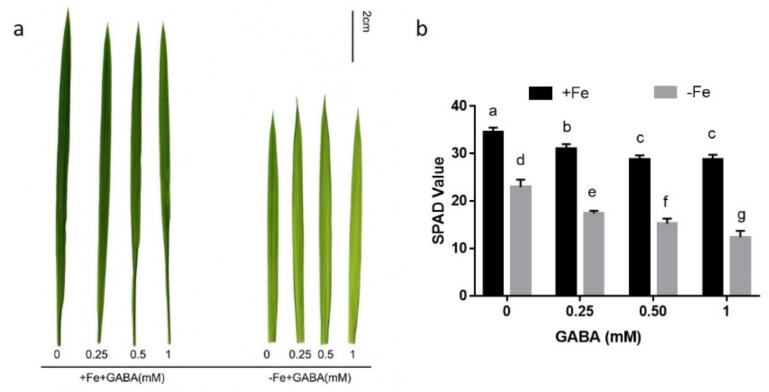
Morphological responses in the fully expanded youngest leaf treated by γ-Aminobutyric acid (GABA) under conditions of Fe-sufficiency (FS) or -deficiency (FD). Two-week-old seedlings were treated with different concentrations of GABA with or without FeSO_4_ (20 μM) for two weeks. (**a**) Photos of leaves in rice seedlings grown in different conditions. (**b**) The soil–plant analysis development (SPAD) value of leaves in rice plants. Data are the means ± SD (*n* = 10). Different letters denote a remarkable difference (*p* ≤ 0.05).

In order to explore the involvement of GABA in the control of Fe homeostasis in rice seedlings, the effects of the GABA (0.25–1 mM) on the chlorophyll concentrations in rice seedlings under conditions of FS and FD were studied. Fe deprivation for two weeks caused apparent chlorosis in newly formed leaves in rice seedlings (Figure 1a). Compared with FS treatment, growth in FD medium reduced the average chlorophyll concentration in the youngest leaves by 33.39%, as determined using a soil–plant analysis development (SPAD) meter (Figure 1b). Application of GABA to seedlings under FD further enhanced leaf chlorosis. Under conditions of FD, 0.5 mM GABA decreased the SPAD value of seedlings from 22.98 to 15.26 (Figure 1b). GABA treatment also decreased the SPAD value under conditions of FS, and the addition of 0.5 mM GABA decreased the SPAD value by 16.52% (Figure 1b). As 0.5 mM GABA had a moderate effect, this amount was used for further testing.

**Figure 2:**
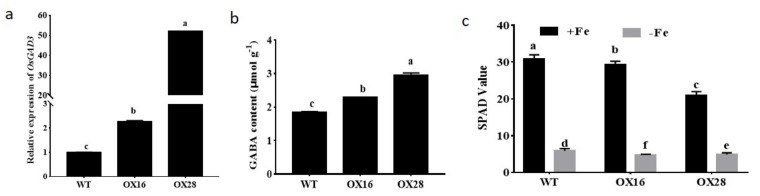
Relative expression of *OsGAD3*, GABA contents and the SPAD Value in wild type and over-expression lines OX16 and OX28. **(a**) Relative expression of *OsGAD3*. (**b**) GABA contents. Data are the means ± SD (n=3). (**c**) The SPAD value in different lines under conditions of FS and FD. Data are the means ± SD (*n* = 10). Different letters denote a remarkable difference (*p* ≤ 0.05).

To investigate if endogenous GABA participates in Fe homeostasis, *OsGAD3,* was overexpressed. The relative expression levels of *OsGAD3* in the two over-expression lines OX16 and OX28 were significantly higher than that in wild type (Nipponbare, NIP) and GABA contents in OX16 and OX28 were respectively 1.24 and 1.59 times of NIP (Figure 2a,b). The two lines were grown in conditions of FS and FD and found that the SPAD values were lower in the youngest leaves of OX16 and OX28 than that of NIP irrespective of Fe supply. Under conditions of FD the SPAD values in OX16 and OX28 were 4.73 and 4.86, respectively, while it was 5.86 in NIP (Figure 2c).

### 2.2. GABA Treatment Suppressed Fe Transportation from Roots to Shoots

**Figure 3:**
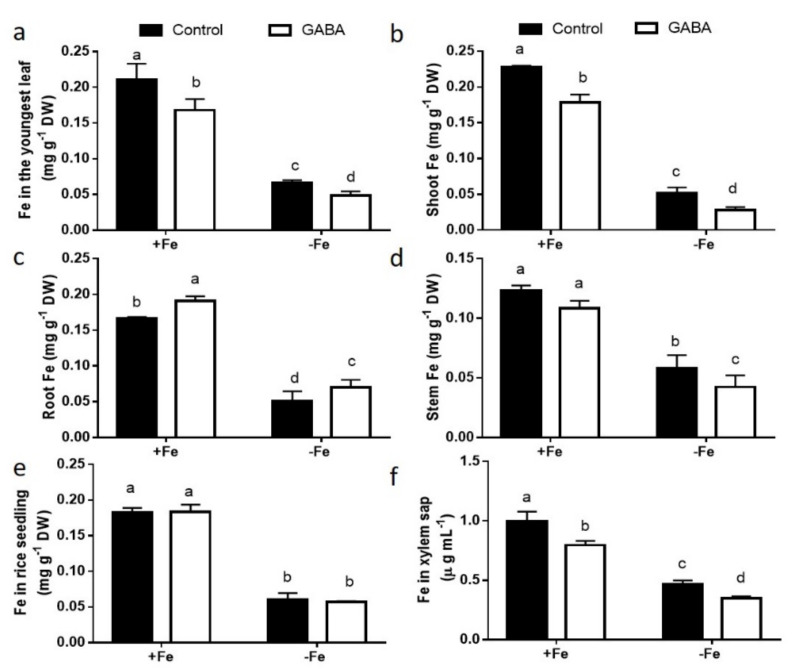
Effect of GABA treatment on Fe concentration. (**a**) Fe in the youngest leaf. (**b**) Fe in shoot. (**c**) Fe in root. (**d**) Fe in stem. (**e**) Fe in rice seedling. (**f**) Fe in xylem sap. The concentration of GABA is 0.5 mM. Treatments and statistical analysis were the same as that in Figure 1.

To explore how GABA contributes to Fe homeostasis, the Fe content of GABA-treated rice seedlings grown under conditions of FS and FD was measured. Compared with FS treatment, FD decreased Fe concentrations in roots and shoots. The application of GABA reduced the Fe concentrations in the youngest leaves, shoots, and stems, but increased the Fe concentration in roots, under conditions of FS and FD (Figure 3). Under conditions of FD, the addition of 0.5 mM GABA led to a 45.83% reduce of the Fe accumulation in shoots and a 27.27% increase in roots compared with the control (Figure 3b,c). Fe level did not differ remarkably between GABA-treated and control whole seedlings, irrespective of Fe supply (Figure 3e). We also found that the Fe content in xylem sap was substantially lesser in GABA-treated than in control seedlings (Figure 3f).

### 2.3. GABA Regulated Epidermis–Pericycle Fe Translocation in Roots

**Figure 4:**
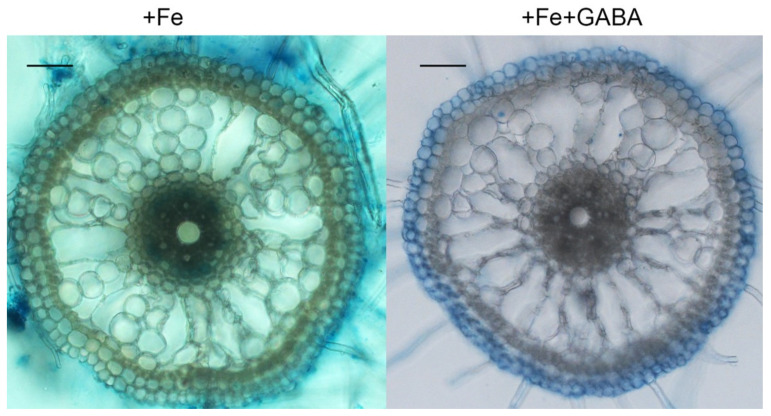
Effect of GABA on ferric accumulation in the roots under conditions of FS. Three-day-old seedlings were treated with 0.5 mM GABA for one week and then roots were stained with Perls’ blue stain to visualize ferric accumulation. Hand-cut sections were observed and pictured. Bars = 50 μm.

Fe^3+^ precipitation in roots under conditions of FS with and without GABA treatment was investigated using Perls’ blue staining [25,26]. The colour in the epidermal cells of roots is darker than that of treated with GABA. In contrast, Fe^3+^ was deposited in the pericycle cells of control roots (Figure 4). These staining results demonstrate that GABA regulates Fe translocation from the epidermis to the pericycle.

### 2.4. GABA Induced Aerenchyma Formation

**Figure 5:**
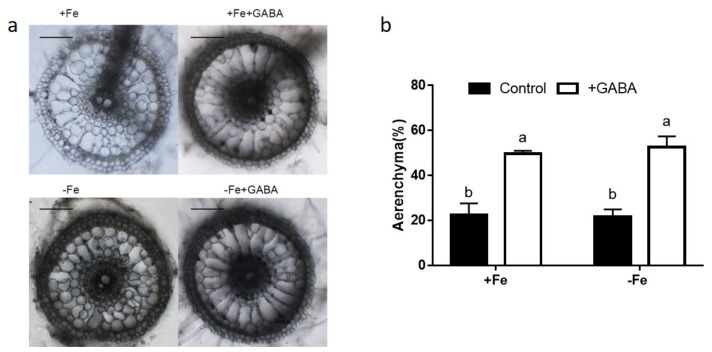
Effect of GABA on the formation of aerenchyma in the roots with or without Fe supply. (**a**) Root sections of seedlings treated with GABA with or without Fe. (**b**) GABA induced aerenchyma formation under conditions of both FS and FD. Three-day-old seedlings were treated with 0.5 mM GABA for one week. Free-hand sections were made 6–8 cm from root tips. Data are averages (± SD) from 5 sections. Bars = 100 μm.

To study the effect of GABA on aerenchyma formation, we determined the percentages of aerenchyma area to cortical cell area in each cross section by software-based analysis of microphotos of root cross sections. Compared with the control, GABA increased the percentages of aerenchyma area by 120.95% under FS conditions (Figure 5). Under conditions of FD, the percentage of aerenchyma in sections of roots treated with GABA was 2.43 times that in controls (Figure. 5).

### 2.5. Transcriptomic Analysis of Roots Treated with GABA Under conditions of FD and FS

To figure out the function of GABA in Fe homeostasis, we investigated the transcriptomes of roots treated with and without 0.5 mM GABA under conditions of FD and FS for 3 days by RNA sequencing (RNA-seq). In total, 88.36 Gb clean data for 12 samples was obtained. The percentage of nucleotides with sequencing quality values ≥ Q30 in each clean data set was ≥92.78%. Samples with 73.79–90.14% clean reads were mapped successfully onto the rice reference genome; 79.95% of total reads in roots was uniquely mapped (Table S1), suggesting that the sequences obtained were of high quality and suitable for subsequent bioinformatics analysis.

**Figure 6:**
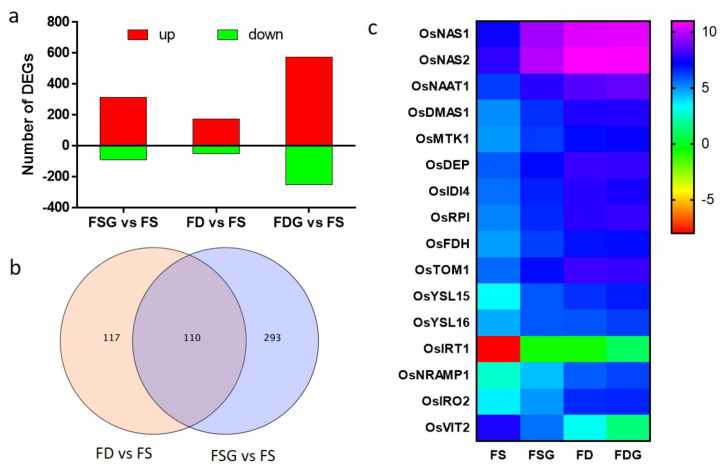
Overview of the changes in transcripts in roots treated with GABA with or without Fe supply. (**a**) Numbers of differentially expressed genes (DEGs) in roots treated with GABA under conditions of Fe-sufficiency (FSG) or -deficiency (FDG) compared to the control (FS). (**b**) Venn diagram of genes regulated by Fe deficiency (FD vs. FS) or GABA (FSG vs. FS) in roots. (**c**) Expression profiles of iron-related genes in roots. The gene -normalized signal intensities are shown in the heat maps using a log2 scale. Two-week-old seedlings were grown in the medium containing 0.5 mM GABA with or without 20 μM FeSO_4_ for three days.

Differentially expressed genes (DEGs) were analyzed in samples treated with and without GABA under conditions of FS and FD and compared with the reference of samples grown under conditions of FS using DESeq software (adjusted *p* ≤ 0.05, fold change ≥ 2). In total, 403 genes were identified as differentially expressed between roots treated with GABA under FS conditions (FSG) and FS. Only 227 DEGs were identified in samples of roots grown under conditions of FDrelative to FS samples. The greatest difference (827 DEGs) was between roots treated with GABA under conditions of FD (FDG) and FS (Figure 6a).

### 2.6. FD-Related Genes Were Induced in GABA-Treated Roots Under Conditions of FS

The analysis of Gene Ontology (GO) enrichment of the DEGs (FSG vs. FS and FD vs. FS) was performed. Six GO terms were remarkably enriched in DEGs (FD vs. FS): transport (GO:0006810), establishment of localization (GO:0051234), localization (GO:0051179), cellular amino acid and derivative metabolic process (GO:0006519), transport activity (GO:0005215), and membrane (GO:0016020). These GO terms were also enriched in DEGs (FSG vs. FS) (Supplementary Figure S1).

We then analyzed genes regulated by both FD and GABA application. In total, 110 genes in roots were regulated by these two treatments (Figure 6b). Among them, 16 genes are involved in Fe acquisition, transportation, or regulation (Figure 6c). Genes such as *OsNAS1*, *OsNAS2*, *OsNAAT1*, and *OsDMAS1* are responsible for MA production and were upregulated in FD and FDG samples (Figure 6c). MAs were produced from SAM and genes involved in methionine cycle were reported to be induced by FD [1,27]. Genes encoding enzymes participating in the methionine cycle, such as methylthioribose kinase 1 (*OsMTK1)*, dehydratase-enolase-phosphatase (*OsDEP*), aromatic aminotransferase (*OsIDI4*), ribose 5-phosphate isomerase (*OsRPI*), and formate dehydrogenase (*OsFDH*), are also induced by both FD and FSG treatments (Figure 6c). The transporter OsTOM1 is involved in DMA secretion into the rhizosphere [5]. The expression levels of OsTOM1 and genes mediated transporting the Fe (III)–DMA complex, such as *OsYSL15* and *OsYSL16* [1,2,7], showed upregulation in FD and FSG samples (Figure 6c). The transcript levels of genes mediating Fe^2+^ transport in the plasma membrane, including *OsIRT1* and *OsNRAMP1* [11,12,13], were upregulated in roots treated with GABA or under conditions of FD (Figure 6c). OsIRO2, a positive regulator of various genes related to Fe uptake [28], was upregulated in FD and FSG samples (Figure 6c). OsVIT2, reported to transport Fe across the tonoplast into the vacuole [29], was down-regulated in FD and FSG samples (Figure 6c).

To validate the RNA-seq data, four Fe-related genes (*OsNAAT1*, *OsNAS1*, *OsDMAS1*, and *OsIRO2*) were selected for qRT-PCR measurement of transcripts from roots three days after treatment. All four genes showed the same trends in RNA fold changes measured by both methods (Supplementary Figure S2), supporting the reliability of the RNA-seq data.

## 3. Discussion

Increasing evidence demonstrates the role of GABA as a signaler controlling growth and development and the responses to abiotic stresses in plant [14,15,17,30]. However, there are few reports on the function of GABA in the control of nutrient uptake and translocation. Previously, we reported that 3 mM GABA significantly reduced ammonium toxicity in rice seedlings [24]. Sheteiwy et al. [31] reported that 0.5 mM GABA alleviated oxidative injury caused by salinity in rice seedlings. In the present study, GABA (0.25–1 mM) was used to study the function of GABA in Fe homeostasis in rice seedlings.

FD is known to cause characteristic leaf chlorosis in plants, as Fe is required for the biosynthesis of chlorophyll [1,2]. Exogenous GABA treatment caused leaf chlorosis under conditions of FS and enhanced the symptom of FD-induced leaf chlorosis (Figure 1), suggesting that GABA participated in the control of Fe homeostasis. In contrast to our result, Guo et al. [32] recently reported that foliar application of 20 mM GABA increased resistance to FD in cucumber. We speculate that this discrepancy is due to difference of species. Rice utilizes the strategy Ⅱ mechanism to uptake Fe, while cucumber plants uptake Fe by strategy I [1]. Furthermore, overexpression of *OsGAD3* gene, encoding a crucial enzyme of GABA production, elevated GABA level and caused lower chlorophyll concentration than wild type under conditions of both FS and FD (Figure 2). These results suggest that GABA is involved in the regulation of Fe homeostasis.

Furthermore, we found that GABA reduced the Fe accumulation in shoots and xylem sap, but elevated the Fe concentration in roots, under conditions of FS and FD (Figure 3). We also found that GABA treatment did not affect Fe content in the whole seedling compared with the control (Figure 3), suggesting that GABA may not increase Fe absorption from soil, but regulates Fe transportation from roots to shoots. In accordance with the increased Fe concentration in roots treated with GABA, we found that more Fe^3+^ was deposited in the epidermal cells of GABA-treated roots than in control roots, in which Fe^3+^ accumulated in the pericycle cells using Perls’ blue staining (Figure 4). This result suggests that more Fe^2+^ was oxidized into Fe^3+^ in the epidermal cells of GABA-treated roots, as FeSO_4_ was supplied in this study. Fe^2+^ is more soluble than Fe^3+^, but presents a risk of oxidative damage in plants when it accumulates at higher concentrations [3]. However, Fe^3+^ often presents as a constituent of oxides and hydroxide polymers and is not useful for plants due to its low solubility [3]. These results suggest that GABA treatment may cause the oxidation of soluble Fe^2+^ into insoluble Fe^3+^ in the epidermal cells of roots and inhibit the transportation of Fe from the epidermis to the pericycle, thereby inhibiting the transportation of Fe from roots to shoots. The mechanism needs to be further studied.

In total, 110 genes were found to be regulated by both GABA and FD. These genes include those related to MA biosynthesis (e.g., *OsNAS*, *OsNAAT*, and *OsDMAS*), the methionine cycle (e.g., *OsMTK1*, *OsDEP*, and *OsFDH*), Fe acquisition and translocation (e.g., *OsTOM1*, *OsYSL15*, and *OsYSL16*)*,* and the regulation of Fe homeostasis (e.g., *OsIRO2* and *OsVIT2*) (Figure 6). These genes are regulated by FD as described previously [1,2,9,33,34]. Consistently, GO analysis found that six GO terms were enriched in DEGs of both FSG vs. FS and FD vs FS (Figure S1). These results show that GABA application under FS conditions induced the upregulation of many FD–related genes, suggesting that GABA application under FS conditions causes FD. These findings further support the concept that GABA is a crucial regulator of Fe homeostasis.

Compared with controls, rice roots treated with GABA showed more aerenchyma formation, irrespective of Fe supply (Figure 5). The aerenchyma allows O_2_ translocation from shoots to roots and releases O_2_ to the rhizosphere [35]. The increased oxygen level triggered more Fe^2+^ oxidization to Fe^3+^ in epidermal cells [36]. Our results show that GABA application induced aerenchyma formation and resulted in Fe^3+^ production in roots. It also lowered the bioavailability of Fe in roots and subsequently decreased the transportation of Fe from the epidermis to the pericycle. Previous studies reported that ethylene (ETH) and reactive oxygen species (ROS) participated in aerenchyma formation in rice roots [37]. GABA was reported to induce ETH production [38] and GABA can reduce ROS accumulation by increasing activities of antioxidant enzymes under various stresses [31]. However, *SIGABA-Ts-* silenced tomato plants increased GABA level and enhanced accumulation of ROS under salt stress [39]. Therefore, it will be interesting to measure ETH and ROS levels in roots and to clarify if ETH and ROS participated in GABA-induced aerenchyma formation in the future research.

GABA content increases three to four times within a few hours under anoxic conditions [14,40]. However, Zhang et al. [41] reported that the tolerance of barley to waterlogging is related to more rapid aerenchyma formation, but is not related to increased GABA content in roots. This result is contrary to our finding that GABA induces more aerenchyma formation in rice. This discrepancy may be due to the examination of different species; rice constitutively develops aerenchyma in roots, whereas barley does not develop aerenchyma under well-drained conditions [42].

Excessive amounts of Fe^2+^ may catalyze the generation of active oxygen species and cause damage to plants [3,36,43]. The exclusion of Fe from the roots is a major adaptive mechanism employed by some plants, which develop extensive aerenchyma and oxide excess Fe^2+^ to Fe^3+^ in the rhizosphere [3,36]. In this study, we found that GABA can induce aerenchyma formation and inhibit Fe transportation from roots to shoots. Therefore, GABA may be involved in the response of rice to excess Fe^2+^.

## 4. Materials and Methods

### 4.1. Plant Materials and GABA Treatment

Rice (*Oryza sativa* L. cv. Huaixianggeng) seeds were imbibed in distilled water for 3 days and then pre-cultured hydroponically with Kimura B solution containing 20 μM FeSO_4_. Seedling (15 days old) were grown in the Kimura B solutions with or without GABA (0.25, 0.5, and 1mM) under FS or FD conditions. FD was initiated on day 15 after germination by omitting 20 μM FeSO_4_ from the culture medium. The seedlings were cultivated in a chamber under 16-h light and 8-h dark at 26 °C. The culture medium was changed every 3 days, and the seedlings were harvested after 2 weeks. The experiments were repeated three times with three replicates each.

### 4.2. Rice Transformation

The OsGAD3 (LOC_Os03g13300) coding sequence was amplified and cloned into the pBWA(V)HU, a binary expression vector with a ubiquitin promoter. The fusion vector was transformed into calli of NIP by Agrobacterium-mediated transformation [44]. The overexpression transgenic plants were identified according to the results of PCR using primers (5′- CATACGCTATTTATTTGCTTGG-3′ and 5′- CTCGTCGTTGATGATCTGGT-3′).

### 4.3. Determination of GABA

Shoot samples were ground using a chilled pestle and mortar in liquid nitrogen, and GABA was extracted according to Kim et al. [45]. GABA was analyzed by an automated amino acid analyzer (Hitachi L-8900, Hitachi High-Technologies Corp., Tokyo, Japan).

### 4.4. Analysis of Xylem Sap

Xylem sap was collected and analyzed two weeks after treatment. The xylem sap was collected as described previously [25]. The concentration of Fe was determined using inductively coupled plasma-mass spectrometry (NexION 300D; PerkinElmer Inc., Shelton, CT, USA).

### 4.5. Analysis of Chlorophyll Content

SPAD values in the youngest leaves were detected with a portable chlorophyll meter (SPAD-502; Minolta Camera Co., Osaka, Japan).

### 4.6. Detedmination of Fe Concentration

Samples were collected two weeks after treatment and then dried and digested as described previously [46]. Fe was measured by inductively coupled plasma-optical emission spectrometry (Optima 8000; PerkinElmer Inc., Shelton, CT, USA).

### 4.7. Perls’ Blue Staining

Seeds were germinated in distilled water for 3 days and then placed into Kimura B solution with or without 0.5 mM GABA under conditions of FS. Perls’ blue staining was performed as described previously [25]. One week after treatment, the roots were immersed in the staining solution and vacuum infiltrated for 15 min. The roots were washed with water, and hand-cut sections were then observed and photographed under an optical microscope (Olympus Optical Co. Ltd., Tokyo, Japan).

### 4.8. Analysis of Aerenchyma in Roots

Freehand cross sections were made with a razor blade 6–8 cm from the primary root tips of seedlings at 7 days after treatment. Sections were pictured with an optical microscope. Aerenchyma formation was estimated by the percentage of cross section possessed by aerenchyma [37]. The areas were measured with Image J (ver. 1.52s).

### 4.9. RNA-seq and Analysis

Total RNA was extracted from the roots 3 days after treatment. This experiment has three biological replicates. The samples were sent to the BMK Company (Beijing, China) for transcriptome sequencing using the Illumine HiSeq2500 platform. Removing low-quality reads, reads containing adapters, and reads containing Poly-N from raw data to obtain clean date. The clean reads were mapped to the rice reference genome IRGSP1.0. Gene expression levels were expressed as fragments per kilobase of transcript per million fragments mapped. DEGs were identified using DESeq. Using the Benjamini and Hochberg approach to adjust *p* values for control of the false discovery rate (FDR). Genes with FDR < 0.05 and log2 (fold change) ≥ 1 were considered as significantly differential expressed. The analysis of Gene Ontology enrichment of DEGs (FSG vs. FS and FD vs. FS) was conducted by agriGO v2.0 [47].

### 4.10. Quantitative Real-Time RT-PCR

Roots were ground into powder in the presence of liquid nitrogen for total RNA extraction. RT-qPCR was performed as described previously [48]. Actin was used as an internal control to normalize the data. The primers for qRT-PCR in this study were referred to previously report [49].

### 4.11. Statistical Analysis

Statistical analyses were conducted using SPSS (ver. 17.0; SPSS Inc., Chicago, IL, USA). remarkable differences between treatments were identified by *p* ≤ 0.05.

## 5. Conclusions

**Figure 7:**
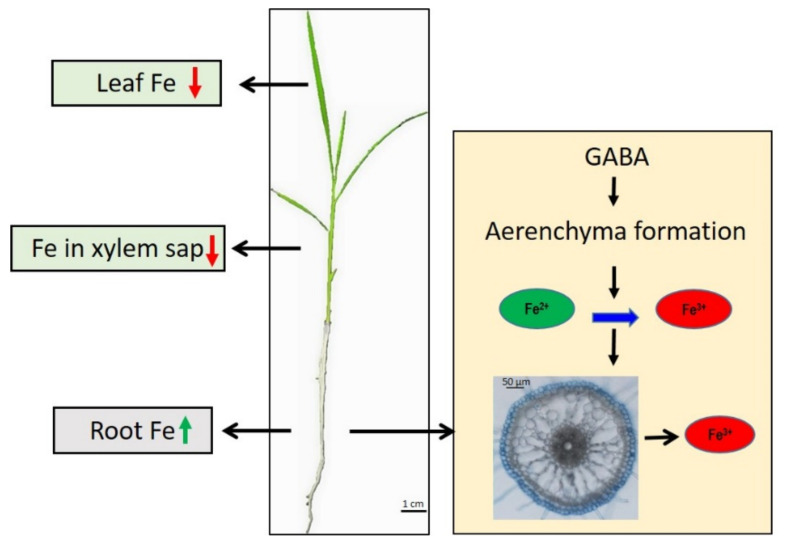
A proposed model for GABA-mediated Fe homeostasis in rice seedlings. GABA treatment induced aerenchyma formation in roots and oxidized soluble Fe^2+^ into insoluble Fe^3+^ and Fe^3+^ was precipitated in epidermal cells of roots, and therefore GABA inhibited the transportation of Fe from roots to shoots. The green arrow denotes increase of Fe content and the red arrows denote decrease of Fe content. The blue arrow denotes Fe^2+^ was oxidized into Fe^3+^.

In conclusion, we demonstrate that GABA is involved in the mediation of Fe homeostasis in rice seedlings (Figure 7). GABA treatment induced aerenchyma formation, the oxidization of soluble Fe^2+^ into insoluble Fe^3+^, and Fe^3+^ precipitation in the epidermal cells of roots. GABA inhibited Fe translocation from the epidermis to the pericycle in roots and subsequently decreased the transportation of Fe from roots to shoots, thereby reducing the Fe content in shoots. These findings will be useful for the mediation of Fe homeostasis in rice seedlings.

## Data Availability

The data presented in this study are available in request from the corresponding author.

## Supplementary Materials

Supplementary materials can be found at https://www.mdpi.com/1422-0067/22/1/220/s1.

## Abbreviations

GABA	γ-Aminobutyric acid
Fe	Iron
GAD	Glutamate decarboxylase
IRT	Iron-regulated transporters
MA	Mugineic acid
YSL	Yellow stripe/yellow stripe 1-like
NAS	Nicotianamine synthase
NAAT	Nicotianamine aminotransferase
DMAS	Deoxymugineic acid synthase
DMA	Deoxymugineic acid
RNA-seq	RNA sequencing
DEGs	Differentially expressed genes
FS	Fe-sufficiency
FSG	with GABA under Fe-sufficient conditions
FD	Fe-deficiency
FDG	with GABA under Fe-deficient conditions
