# Supplementary material for: γ-Aminobutyric Acid Suppresses Iron Transportation from Roots to Shoots in Rice Seedlings by Inducing Aerenchyma Formation
Source: Int J Mol Sci. 2020 Dec 28;22(1):220. doi: 10.3390/ijms22010220 (PMC7795648; doi:10.3390/ijms22010220)
Supplement: Supplementary file 1 [file ijms-22-00220-s001.pdf]

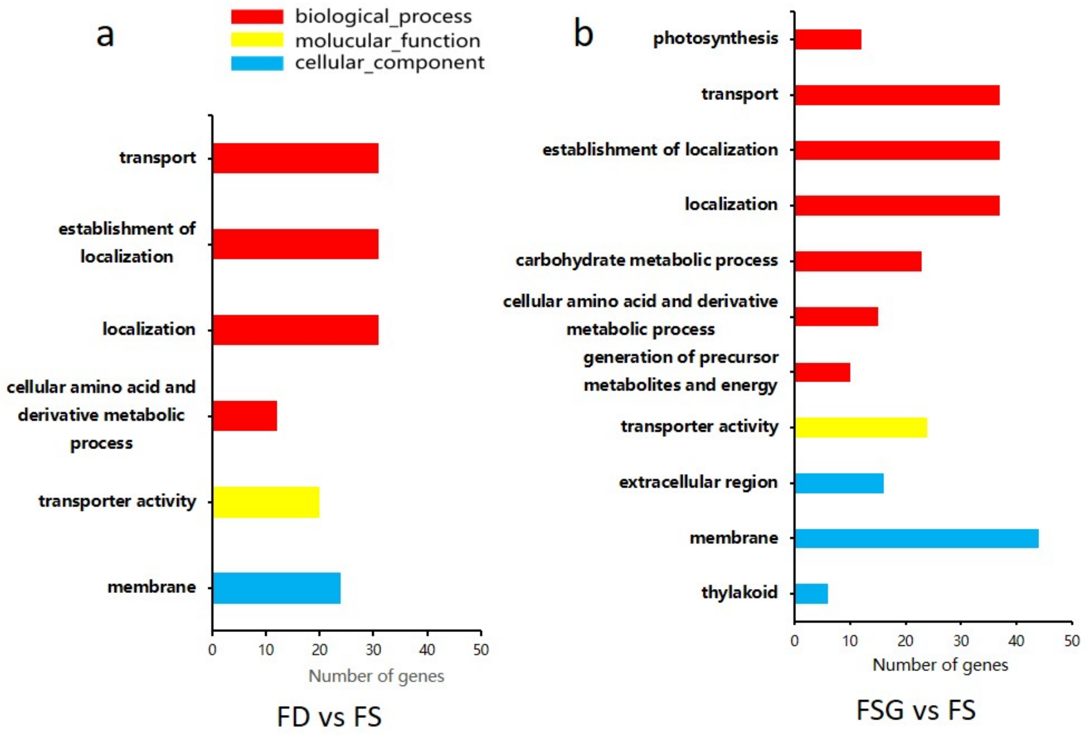

Supplemental Figure S1. Gene Ontology analysis using agriGO v2.0 reveals enrichment of DEGs. (a) DEGs of FD vs FS. (b) DEGs of FSG vs FS.

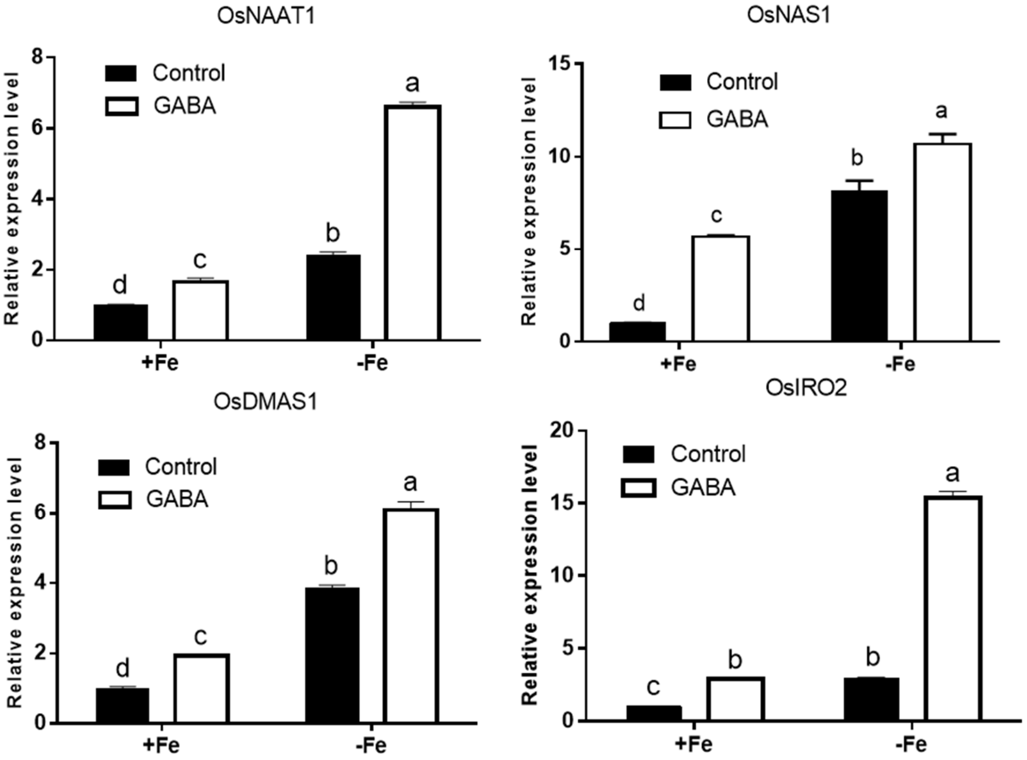

Supplemental Figure S2. Relative expression of iron-related genes in roots by qRT-PCR

Two-week-old seedlings were transferred to the solutions containing 0.5 mM GABA with or without 20 μm $FeSO_4$ for three days. Actin was used as an internal control to normalize data.

Supplemental Table 1 Information for RNA-seq production for the 12 samples

Samples	Clean bases	GC Content	%≥Q30	Total Reads	Mapped Reads	Uniq Mapped Reads	Multiple Map Reads
RF-1	7,414,828,532	52.80%	93.47%	49,791,302	42,650,853 (85.66%)	41,602,819 (83.55%)	1,048,034 (2.10%)
RF-2	6,301,244,686	53.81%	93.64%	42,483,984	37,826,873 (89.04%)	36,911,574 (86.88%)	915,299 (2.15%)
RF-3	6,070,169,088	52.51%	93.01%	40,655,326	32,603,277 (80.19%)	31,806,309 (78.23%)	796,968 (1.96%)
RFG-1	6,659,897,974	53.91%	92.99%	44,736,922	36,961,824 (82.62%)	35,819,519 (80.07%)	1,142,305 (2.55%)
RFG-2	7,341,018,652	53.09%	93.23%	49,345,088	39,593,117 (80.24%)	38,280,180 (77.58%)	1,312,937 (2.66%)
RFG-3	8,750,736,314	52.99%	93.58%	58,787,418	47,381,878 (80.60%)	45,800,233 (77.91%)	1,581,645 (2.69%)
RDF-1	8,907,770,450	53.46%	93.38%	59,806,722	50,488,024 (84.42%)	49,164,303 (82.21%)	1,323,721 (2.21%)
RDF-2	6,787,410,118	53.45%	93.30%	45,541,044	39,971,694 (87.77%)	38,948,773 (85.52%)	1,022,921 (2.25%)
RDF-3	6,709,554,942	54.42%	93.39%	45,105,784	40,657,362 (90.14%)	39,584,990 (87.76%)	1,072,372 (2.38%)
RDFG-1	9,483,351,916	51.98%	92.78%	63,609,292	47,242,895 (74.27%)	45,702,794 (71.85%)	1,540,101 (2.42%)
RDFG-2	7,035,429,974	52.89%	93.89%	47,369,288	34,956,023 (73.79%)	33,848,144 (71.46%)	1,107,879 (2.34%)
RDFG-3	6,894,835,308	53.92%	93.85%	46,286,404	36,516,331 (78.89%)	35,325,362 (76.32%)	1,190,969 (2.57%)

Note: RF: +Fe; RFG: +Fe+GABA; RDF: -Fe; RDFG: -Fe+GABA
